# Hysteresis branch crossing and the Stoner–Wohlfarth model

**DOI:** 10.1038/s41598-020-72233-x

**Published:** 2020-09-15

**Authors:** Scott A. Mathews, Alexander C. Ehrlich, Nicholas A. Charipar

**Affiliations:** 1 Materials Science and Technology Division, Naval Research Laboratory, Washington, DC 20375 USA; 2 Leidos Inc., 4001 Fairfax Dr., Arlington, VA 22203 USA

**Keywords:** Materials science, Physics

## Abstract

The Stoner–Wohlfarth model predicts the crossing of the ascending and descending branches of the hysteretic magnetization curve. This crossing behavior has widely been dismissed, with the claim that it violates the laws of thermodynamics. Experimental verification of hysteresis branch crossing has not been acknowledged in the literature. Here we show, both theoretically and experimentally, that the crossing of the ascending and descending branches of the magnetization curve is a robust, reproducible phenomenon which does not violate any fundamental law.

## Introduction

The Stoner–Wohlfarth model^1^, first published in 1948, describes the magnetic behavior of anisotropic, ferromagnetic particles. In particular, the Stoner–Wohlfarth model applies to non-interacting, magnetic particles which change their state of magnetization by coherent rotation, rather than domain wall motion. This widely used model predicts the crossing of the ascending and descending branches of the magnetization curve when the applied field is close to the magnetic hard-axis. However, experimental observation of this hysteresis branch crossing has not been acknowledged in the literature. In fact, the crossing of hysteresis branches has been labelled as being nonphysical^2^ and a problem^3^ with the Stoner–Wohlfarth model. Here we show that the crossing of the ascending and descending branches of the magnetization curve does occur and is consistent with the Stoner–Wohlfarth model. We found that the crossing of hysteresis branches in the Ni:LiNbO_3_ system (thin film nickel on lithium niobate) is a robust and reproducible phenomenon. Contrary to previous reports, we show that the crossing of hysteresis branches is physically realistic^4^ and violates no fundamental law. Additionally, we note that several previously published reports^5–7^ appear to show the crossing of hysteresis branches in their data under similar conditions without providing an explanation of the phenomenon. Our results demonstrate that the crossing of hysteresis branches must be considered when analyzing or modelling the magnetic behavior of anisotropic, ferromagnetic materials that undergo coherent rotation. Given the recent interest in anisotropic magnetic materials in the fields of spintronics^8^ and nanomagnetic logic^9^, we anticipate that the crossing of hysteresis branches may influence the understanding and development of magnetic devices for sensing, data storage, and nanomagnetic computing.

The Stoner–Wohlfarth (SW) model calculates the magnetic free energy for a single, uniaxial particle, based on the anisotropy energy and the Zeeman energy. Using a zero temperature approximation, the model allows the calculation of the magnetization as a function of the magnitude and direction of the applied field.

**Figure 1:**
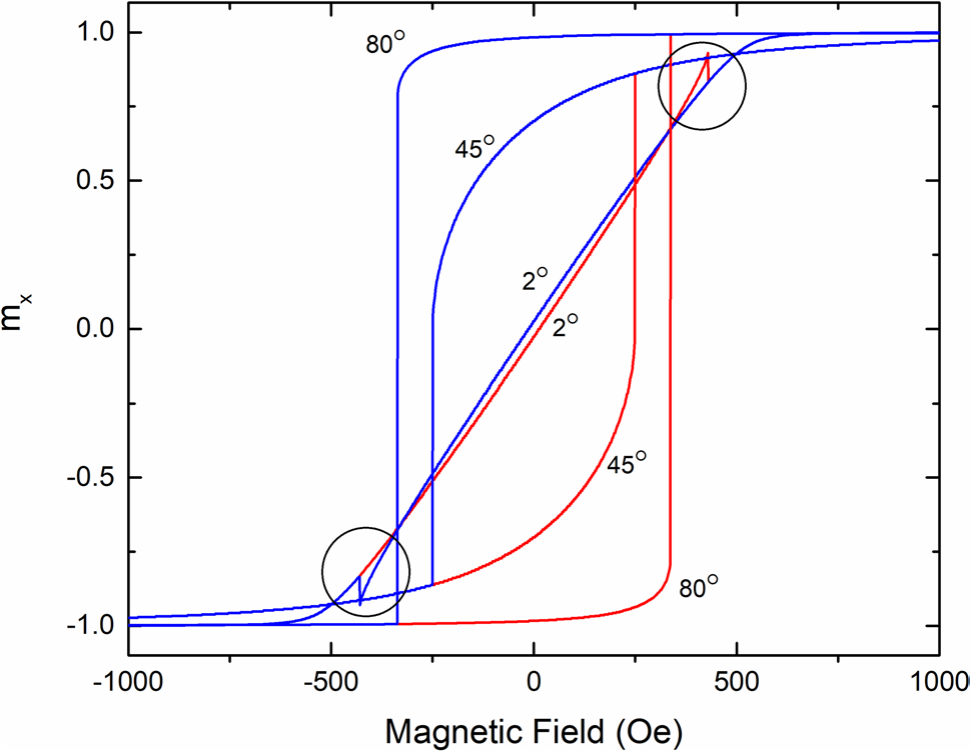
The reduced, x-component of the magnetization (m_x_ = M_x_/M_S_) as a function of applied field calculated using the SW-model with the field at 2°, 45°, and 80° from the hard axis. The ascending curves are shown in red, while the descending curves are shown in blue. The hysteresis branch crossing (HBC) is clearly visible in both the ascending and descending branches at 2°. Black circles highlight the HBC’s.

Hysteresis curves for both components (parallel and perpendicular to the applied field) of the magnetization can be generated numerically for a SW-particle. We have implemented an algorithm to generate numerical solutions to the SW-model, based on the values of the saturation magnetization (M_s_) and the anisotropy field (H_k_) of the simulated particle, and the angle of the applied field with respect to the hard axis (α). Supplemental Section S1 gives the details of the numeric simulation of the zero temperature SW-model. Figure 1 shows the results of this simulation for an anisotropy field of H_k_ = 500 Oe at three different angles of applied field with respect to the hard axis. It is clear from Fig. 1 that the SW-model predicts the crossing of the ascending and descending branches of the magnetization when the applied field is close to the hard axis. The hysteresis branch crossing was noted by Stoner and Wohlfarth in their original paper^1^, however, very little discussion was provided.

In order to more accurately simulate a real particle, we have implemented an algorithm to numerically generate solutions to a temperature dependent SW-model. To account for thermal fluctuations, the procedure outlined by Lanci and Kent^10^ is followed. This algorithm assumes that when two energy minima are present in the free energy landscape, the equilibrium populations of the two minima are dictated by Boltzmann statistics. In order to calculate the energy landscape in real units, such that the magnetic free energy can be compared to the thermal energy (k_B_T), the finite temperature algorithm requires a parameter representing the effective particle volume (V_eff_), or the volume over which the exchange interaction acts. The height of the energy barrier between the two states is calculated from the energy landscape and the approach-to-equilibrium equation is solved for an appropriate measurement time to yield the fractional population of magnetization in the two states (n_1_ and n_2_). The net magnetization is calculated as the weighted sum of the two states. Supplemental Section S2 gives the details of the numeric simulation of the finite temperature SW-model.

The algorithm implementing the finite temperature SW-model can then be extended to simulate a distributed anisotropy. Whereas the original SW-model assumes a unique orientation and magnitude of the anisotropy field, a more realistic simulation must account for a finite distribution of anisotropy orientation and magnitude. Any physical sample, comprising an ensemble of particles, must have some finite variation in anisotropy from particle to particle. In order to better model such a physical sample, the finite temperature SW-model algorithm was used to simulate the magnetization of a SW-particle over a range of anisotropy magnitudes, and the behavior of the ensemble was calculated as the weighted sum of the individual simulations, assuming a normal (Gaussian) distribution with a standard deviation of σ_Hk_. Measurements of the transverse remanence of the samples in this study indicated that angular distribution of the anisotropy was less than 0.5° FWHM. As a result, subsequent simulations ignored the angular (orientation) distribution and modeled only a distributed magnitude of anisotropy field. Supplemental Section S3 gives the details of the transverse remanence measurement.

## Results

### Simulation

**Figure 2:**
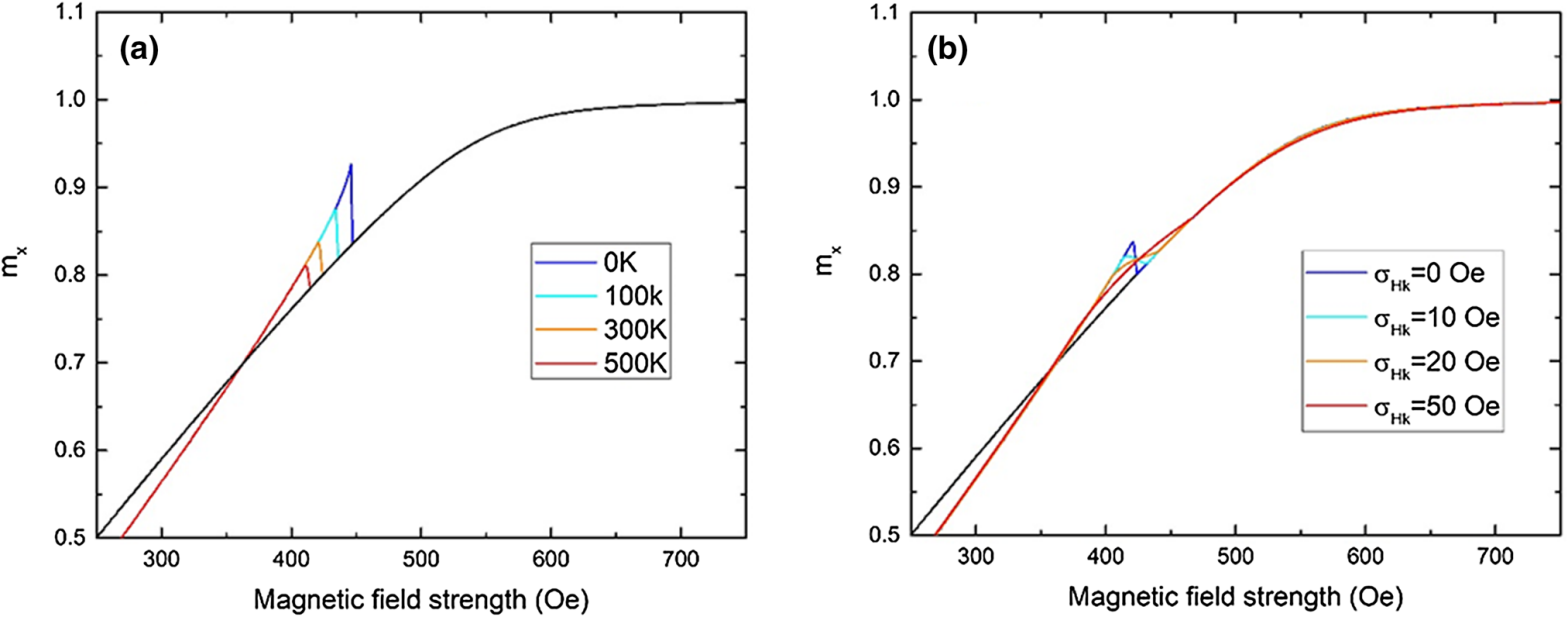
The reduced, x-component of the magnetization as a function of applied field calculated using the finite temperature SW-model for an angle of α = 2°. Only the first quadrant of the MH-curve is shown for clarity. Ascending branches are shown in color, while the descending branch is shown in black. (**a**) Variable temperature with a unique value of anisotropy. (**b**) Variable anisotropy width for a temperature of 300 K.

Simulations were performed for a SW-particle with a unique anisotropy field at several different temperatures. The hysteresis branch crossing (HBC) is still present in the simulated magnetization curves, provided the temperature is sufficiently low. The effect of increased temperature is to reduce the area of the HBC and to move the HBC to lower values of applied field. This effect is demonstrated in Fig. 2a, which shows the simulated magnetization curves for four different temperatures with the field applied 2° from the hard axis. Note that the descending branch of the magnetization, shown in black, is not effected by temperature.

Additional simulations were performed for a SW-particle at fixed temperature and a variable anisotropy distribution width (σ_Hk_). The effect of a distributed anisotropy magnitude is to smooth out the sharp feature of the HBC. While the area of the HBC is found to remain unchanged, the fact that the crossed region is “smeared out” makes it less obvious. This effect is demonstrated in Fig. 2b, which shows the magnetization curves for four different anisotropy distribution widths with the field applied 2° from the hard axis, and a temperature of 300 K. Note that the descending branch of the magnetization, shown in black, is not significantly affected by the distributed anisotropy. The numeric parameters used in the simulations are given in Table S4.1 in the Supplemental Section.

### Experimental

**Figure 3:**
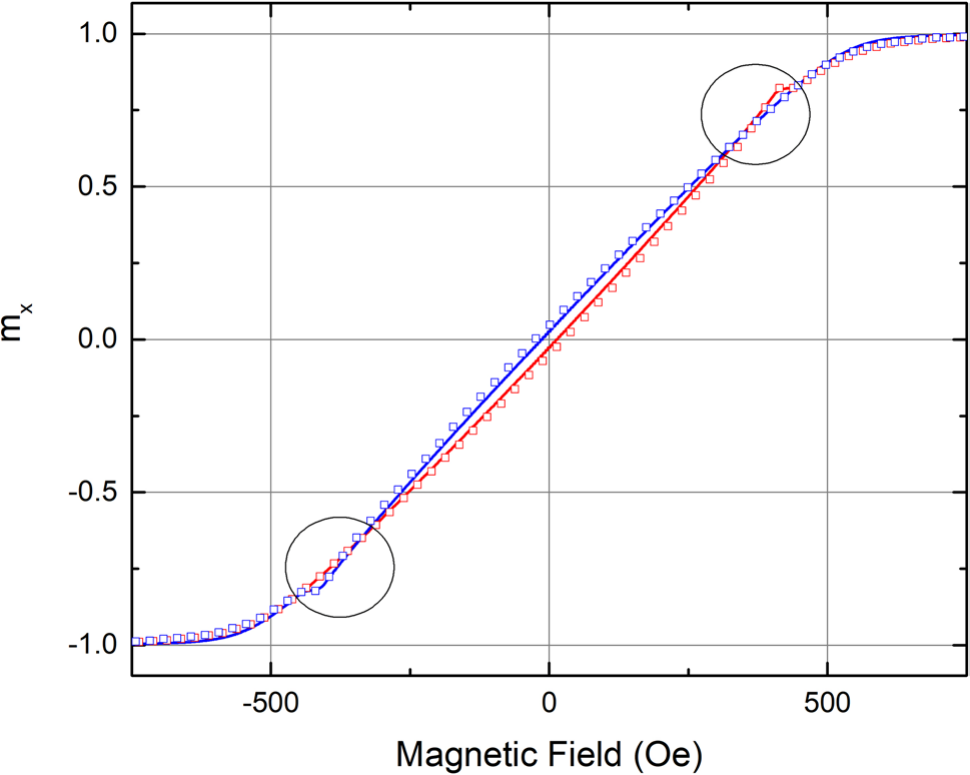
The reduced x-component of the magnetization as a function of applied field for an angle of α = 2°. Open squares represent the measured data, while solid lines represent the simulation. Ascending and descending curves are shown in red and blue, respectively. Black circles highlight the HBC.

**Figure 4:**
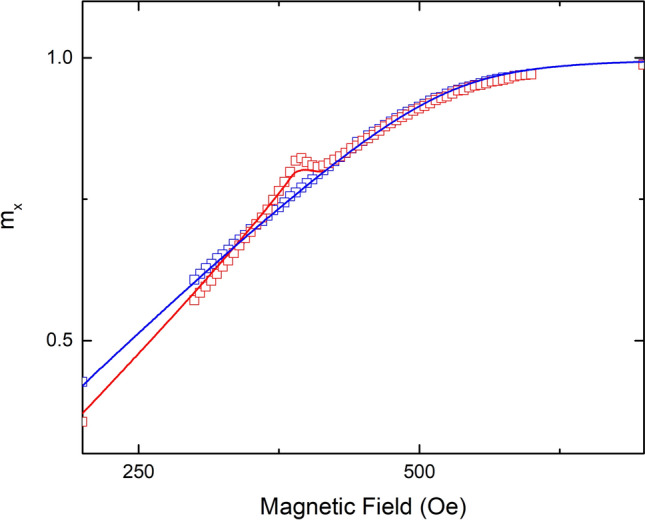
The reduced, x-component of the magnetization as a function of applied field for an angle of α = 2° and a field step of 5 Oe in the region of interest. Squares represent the measured data and solid lines represent the simulation. Ascending and descending curves are shown in red and blue, respectively. Only a portion of the MH-curve is shown for clarity.

Nickel thin films on LiNbO_3_ were annealed at low temperature and measured by vibrating sample magnetometery (VSM). Details are provided in the “Methods” section below. The low temperature annealing creates a uniaxial magnetic anisotropy in the film due to the mismatch of coefficient of thermal expansion between film and substrate^11^. All samples in this study demonstrated HBC consistent with the finite temperature SW-model when the angle of the applied field was in the range 0.5° <|α|< 5° from the hard axis. Figure 3 shows the measured and simulated x-component of the magnetization for a particular sample with field applied 2° from the hard axis. The HBC’s (highlighted with black circles) are clearly visible in both the ascending and descending branches. The results shown in Fig. 3 are representative of all samples in this study. As seen in Fig. 3, the HBC occurs over a narrow range of applied field and is just barely resolved in the measured data when using a field step of 25 Oe. Subsequent measurements of the x-component of the magnetization were performed on a different VSM using a 5 Oe field step, in order to more accurately resolve the HBC. Figure 4 shows the measured (open squares) and simulated (solid lines) x-component of the magnetization for the same sample with the applied field 2° from the hard axis and a field step of 5 Oe in the region of interest. Agreement between the simulation and the measured data is excellent, and the HBC is clearly visible.

## Discussion

**Figure 5:**
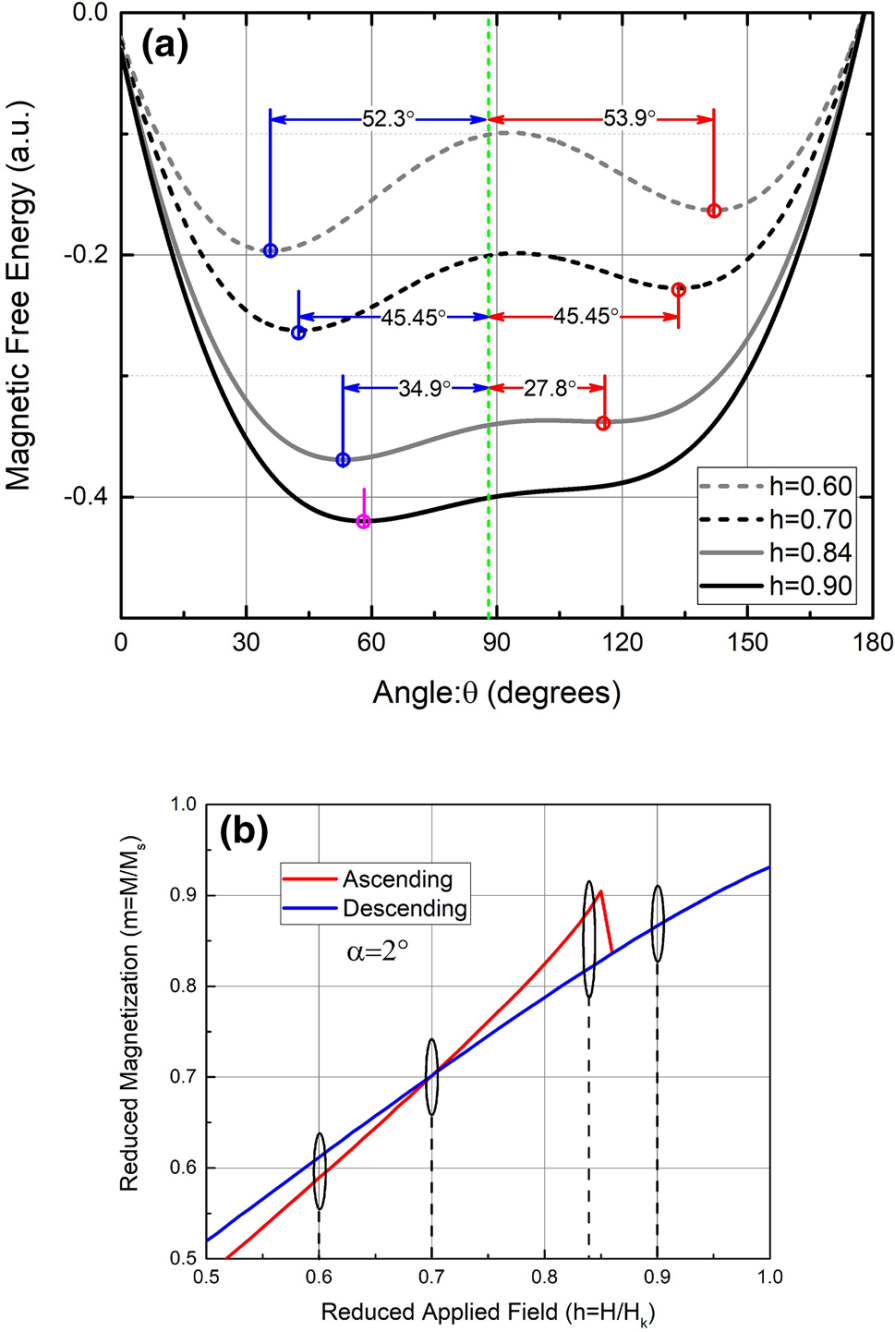
(**a**) The energy landscapes (magnetic free energy vs. theta) for four specific values of field applied at an angle of α = 2° (88° w/respect to the easy axis). The minima in each energy landscape is marked with a circle. The occupied minimum on the ascending and descending branches are shown in red and blue, respectively. The orientation of the applied field is marked by the dashed green line. (**b**) The positive field portion of the hysteresis loop for α = 2°, as calculated by the Stoner–Wohlfarth model. The four field points, corresponding to the energy landscapes in (**a**) are circled: below the crossing, at the crossing point, in the crossed region, and above the crossing.

In order to elucidate the physics behind the HBC, we examine the energy landscape (magnetic free energy as a function of theta) of a Stoner–Wolhfarth particle at four specific values of applied field: below the crossing, at the crossing point, in the crossed region, and above the crossing. These four energy landscapes are shown in Fig. 5a, in the region 0° < θ < 180° as measured with respect to the easy-axis. The four corresponding points on the ascending and descending hysteresis curves are shown in Fig. 5b. The energy landscapes are calculated from the Stoner–Wohlfarth model, assuming the applied field is 2° from the hard axis (θ = 88°). In Fig. 5a, the minima in the energy landscapes are shown by small circles. The minimum that is occupied on the ascending branch is shown in red, while the minimum occupied on the descending branch is shown in blue. When the energy landscape has only one minimum, the minimum is shown by a magenta circle. The orientation of the applied field (and therefore the direction of the measured magnetization) is shown by the dashed, green line.

When the reduced applied field, h = 0.6, the energy landscape has two minima. This curve corresponds to a point on the hysteresis curve below the crossing on the positive side. On the ascending branch of the hysteresis curve, the rightmost minimum (at θ ≈ 142°) is occupied, while the leftmost minimum (at θ ≈ 36°) is occupied on the descending branch. From Fig. 5a), it is seen that the energy minimum, and therefore the orientation of the magnetization, is closer to the direction of the applied field on the descending branch. As a result, the measured component of the magnetization will be higher on the descending branch, as compared to the ascending branch (representing a so-called “normal region” of the hysteresis loop). When the reduced applied field, h = 0.70, the energy landscape again has two minima, equidistant from the direction of the applied field. This point represents the crossing: the point at which the measured magnetization is the same for the ascending and descending branches. When the reduced applied field, h = 0.84, two energy minima exist, however, the occupied minimum on the ascending branch is closer to the direction of the applied field. This represents a point on the hysteresis curve in the crossed region: the component of the measured magnetization is larger for the ascending branch, as compared to the descending branch. When the reduced applied field, h = 0.9, only a single energy minimum exists, and the measured magnetization is the same for both branches of the hysteresis curve. This represents a point above the branch crossing.

It is clear from this analysis that when the applied field is just below the hard axis (for example, applied field at θ = 88°), the energy minima of both the ascending and descending branches move toward the applied field as the field is increased. However, at an applied field greater than h = 0.70, the minimum occupied on the ascending branch moves toward the applied field more rapidly than the descending branch, resulting in the crossing of the branches. If the field were applied at θ = 92° (2° from the hard axis in the other direction, the energy landscapes and the occupied minima would be the mirror image of those shown in Fig. 5a, and the branch crossing would occur in the exact same way.

**Figure 6:**
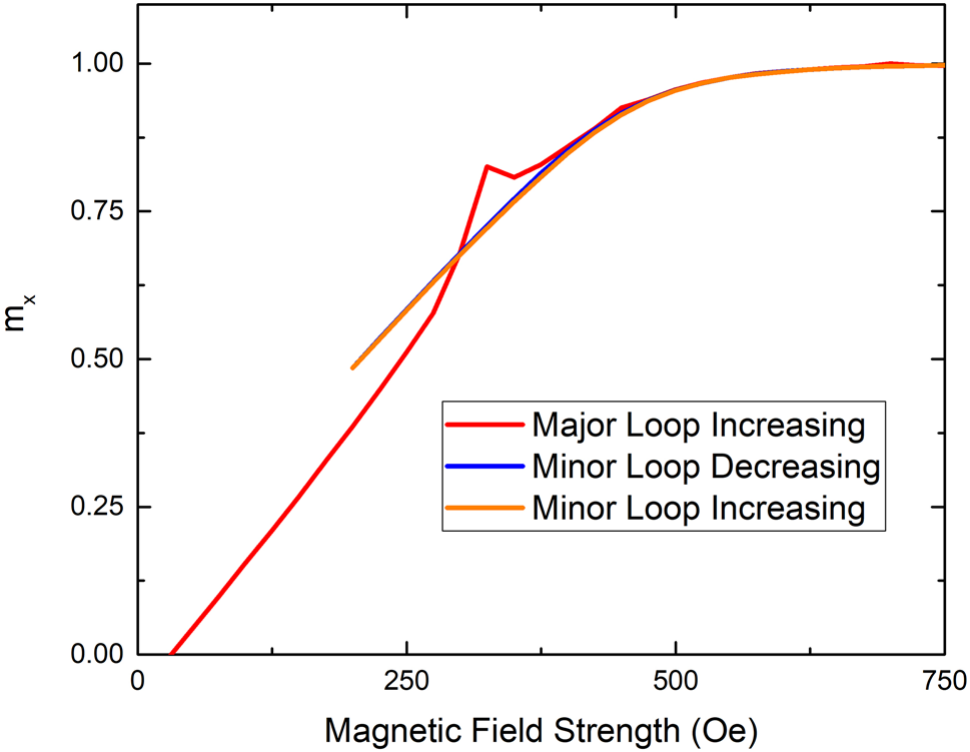
Measured data of the reduced, x-component of the magnetization as a function of applied field for an angle of α = 3°. The red curve (major loop) clearly shows a HBC. The blue and orange curves (representing a minor loop) show no HBC. Only a portion of the MH-curve is shown for clarity.

It is important to note that close examination of Fig. 3 indicates that both the simulated and measured hysteresis loops do not violate the laws of thermodynamics. The area encompassed by the region in which the descending branch is above the ascending branch (the lens shaped, “eye”) in the hysteresis loop represents magnetic energy loss. The area encompassed by the two HBC’s represents an effective energy gain. Because the area enclosed by the two HBC’s is smaller than the area of the eye, the conservation of energy is not violated. Traversing a major hysteresis loop constitutes a net energy loss. Numeric integration of the simulated results confirms that the area enclosed by the two HBC’s is always less than the area of the eye. As pointed out by Hanmin et al*.*^12^, VSM measurements of materials with a strong uniaxial anisotropy are problematic near the hard axis. The process of centering the sample, often referred to as “saddling”, is crucial when attempting to observe the HBC. A poorly saddled (or centered) sample can cause the appearance of an HBC when none actually exists or cause the disappearance of the HBC when it does exist. Similarly, a poorly saddled sample can increase or decrease the area of the eye in a measured hysteresis loop, thereby creating the appearance of a violation of conservation of energy. All samples in this study, when properly saddled, showed measured hysteresis loops with a net energy loss. Because the HBC only occurs when a major loop is traversed, repeatedly traversing the region of the HBC does not result in a net energy gain. This fact is demonstrated experimentally in Fig. 6, which shows the reduced magnetization of a sample with field applied 3° from the hard axis. In Fig. 6, the red curve shows a clear HBC, after having applied a negative saturating field (a major loop). After traversing the HBC in the increasing direction, the field is reduced to a value well below the HBC, but well above negative saturation (blue curve). The field is then increased, so as to traverse the region of the HBC in the increasing direction again (minor loop, shown in orange). The HBC is not present in the minor loop. In fact, the blue curve (decreasing field) and the orange curve (increasing minor loop) effectively lie on top over one another. Therefore, repeatedly traversing the region of the HBC in a minor loop does not result in a net energy gain.

It is widely assumed that hysteresis branches cannot or do not cross^4^. In fact, the crossing of hysteresis branches has been referred to as being energetically incorrect and nonphysical^2^, and a problem^3^ with the SW-model. A number of authors have proposed other models or modifications to the SW-model in order to eliminate the “problem” of crossover^13–15^. It is unclear why many researchers have assumed that HBC is non-physical. It appears that some researchers have rejected the idea because of the erroneous assumption that it violates the laws of thermodynamics (1st or 2nd law). The data presented in this paper clearly indicate that HBC is a real physical phenomenon, is well described by the finite temperature SW-model, and does not violate the laws of thermodynamics.

In addition to the data demonstrating HBC presented in this work, a number of reports in the literature clearly show HBC^5–7,16^ in their data. While many of these previously published works do not call attention to the HBC or provide an explanation for the phenomenon, the figures in these reports show qualitatively similar results to those presented above. As predicted by the SW-model, the HBC’s observed in these reports occur in magnetization measurements performed at or near the hard axis^5–7,16^.

## Methods

Nickel films were deposited by DC magnetron sputtering on polished 128° Y-cut LiNbO_3_ substrates. The nickel layers were between 90 and 100 nm thick, as measured by stylus profilometery. A 2 nm tantalum capping layer was deposited by DC magnetron sputtering, without breaking vacuum, in order to prevent the formation of nickel oxide on the surface. To avoid the effects of in-plane shape anisotropy, samples were patterned into 7 mm disks using conventional photolithography and wet-etch. Samples were thermally annealed under vacuum (< 10^–6^ Torr) at 325 °C for 2 h.

While all data in this report were obtained from a sample processed as stated above, it is worth noting that a large number of samples were produced which showed the same behavior. The HBC was repeatedly observed in samples deposited by e-beam evaporation, as well as sputtering. Samples with and without capping layers and adhesion layers all showed qualitatively the same behavior. Samples deposited on Y-cut LiNbO_3_ (as opposed to 128° Y-cut) and samples annealed at 250 °C showed the HBC, but with significantly different anisotropy fields.

In plane MH-curves were measured at room temperature using two different commercial vibrating sample magnetometers (VSM). The first, a so-called vector VSM, was equipped with both X and Y-axis pick-up coils to allow the simultaneous measurement of the x- and y-components of the magnetization, while the second VSM only allowed the measurement of the x-component of the magnetization. Again, it is worth noting that the phenomenon of HBC was observed in multiple measurement systems, on multiple samples. Removal and reinsertion of the sample had no effect on the HBC.

## Conclusion

We have demonstrated that the crossing of hysteresis branches in highly anisotropic ferromagnetic materials is physical and is well-described by the finite temperature Stoner–Wohlfarth model. This phenomenon, which has been widely ignored for more than 70 years, violates no fundamental law. We find the hysteresis branch crossing in annealed nickel-LiNbO_3_ samples to be robust and reproducible, having been repeatedly observed in more than 20 individual samples, using multiple measurement systems. Although the crossing of magnetic hysteresis branches may be difficult to observe in experiment, we note that a number of previously published reports demonstrate the phenomenon. We conclude that under the appropriate conditions, magnetic hysteresis branches can and do cross. Our results demonstrate that the crossing of hysteresis branches must be considered when analyzing or modelling the magnetic behavior of anisotropic, ferromagnetic materials that undergo coherent rotation.

## Supplementary information

Supplementary Information
