# Author Correction: Hysteresis branch crossing and the Stoner–Wohlfarth model

**DOI:** 10.1038/s41598-021-81864-7

**Published:** 2021-01-28

**Authors:** Scott A. Mathews, Alexander C. Ehrlich, Nicholas A. Charipar

**Affiliations:** 1 Materials Science and Technology Division, Naval Research Laboratory, Washington, DC 20375 USA; 2 Leidos Inc., 4001 Fairfax Dr., Arlington, VA 22203 USA

Correction to: *Scientific Reports* 10.1038/s41598-020-72233-x, published online 15 September 2020

This Article contains an error in the Introduction.

“Additionally, we note that several previously published reports^5,6,7^ appear to show the crossing of hysteresis branches in their data under similar conditions without providing an explanation of the phenomenon.”

should read:

“Additionally, we note that previously published reports^5,6^ appear to show the crossing of hysteresis branches in their data under similar conditions without providing an explanation of the phenomenon. At least one previously published report^7^ shows the crossing of hysteresis branches, and confirms the behavior using micromagnetic simulations.”

