# Supplementary material for: Hysteresis branch crossing and the Stoner–Wohlfarth model
Source: Sci Rep. 2020 Sep 15;10:15141. doi: 10.1038/s41598-020-72233-x (PMC7494899; doi:10.1038/s41598-020-72233-x)
Supplement: Supplementary file 1 — Supplementary Information [file 41598_2020_72233_MOESM1_ESM.pdf]

Supplemental Materials: Hysteresis branch crossing and the Stoner-Wohlfarth model

Scott A. Mathews[*1], Alexander Ehrlich[2], and Nicholas Charipar[1]
[1]Materials Science and Technology Division, Naval Research Laboratory, Washington, DC 20375
[2]Leidos Inc., 4001 Fairfax Dr., Arlington, VA 22203

[*]Corresponding author: scott.mathews@nrl.navy.mil

S1: Zero temperature Stoner-Wohlfarth Model

The in-plane magnetization curves (MH-curves) have been calculated numerically, using the Stoner-Wohlfarth (SW) model [1]. All samples in this work are ferromagnetic thin films, and as a result, shape anisotropy confines the magnetization to the plane of the film. Therefore, only in-plane magnetic behavior is modeled. The terms “hard axis” and “easy axis”, therefore, refer to the in-plane hard and easy axes, respectively. Because the samples are patterned in the shape of a circle, the shape anisotropy in-plane is ignored. Under these conditions, the SW-model can be used to predict the equilibrium orientation of the magnetization (θ_{eq}) as a function of the applied field based on two parameters: the angle between the applied field and the hard axis (α) and the anisotropy field (H_k). The geometry and angular relations are shown in figure S1.1.

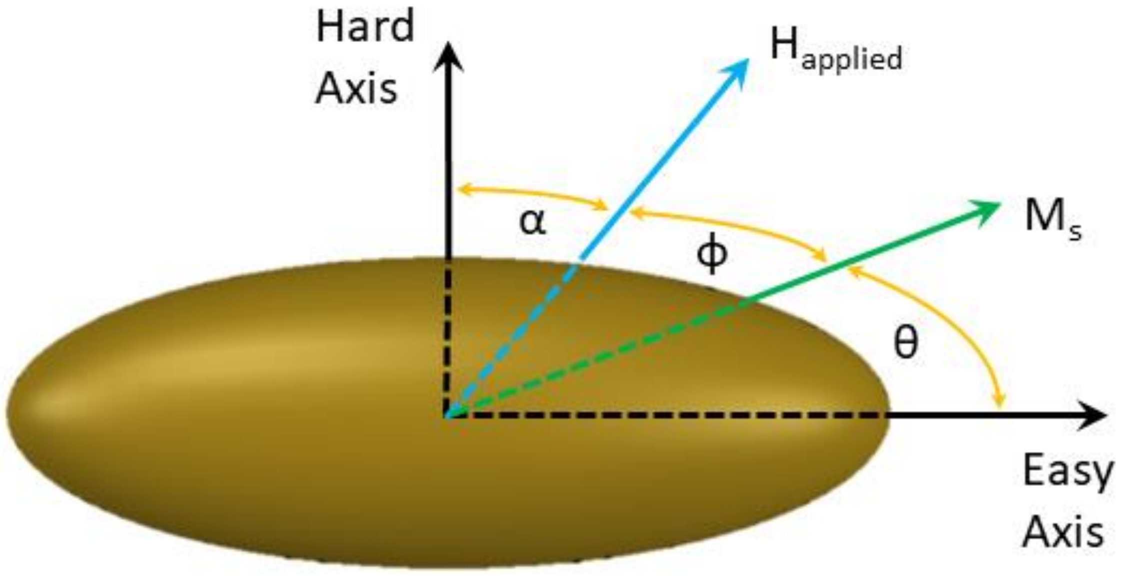

Figure S1.1. Geometry and angular relations for the SW-model.

While figure S1.1 shows an ellipsoidal particle with shape anisotropy, the SW-model ignores the source of the anisotropy and treats only the total resultant anisotropy. The SW-model computes the total magnetic free energy as the sum of two terms: the anisotropy energy and the Zeeman energy. With the geometry given in figure S1.1, these two terms can be written as:

$$E_A = V_{eff} K_u sin^2(\theta) = \frac{V_{eff} H_k M_s}{2} sin^2(\theta) \quad \text{[eq. S1.1]}$$

$$E_Z = -V_{eff} \overrightarrow{M_s} \cdot \overrightarrow{H_a} = -V_{eff} M_s H_a \cos\varphi = -V_{eff} M_s H_a \sin(\theta + \alpha) \quad \text{[eq. S1.2]}$$

where V_{eff} is the effective volume of the particle, K_u is the anisotropy energy, H_k is the anisotropy field, M_s is the saturation magnetization, θ is the angle between the magnetization and the easy axis, H_a is the applied field, and α is the angle between the applied field and the hard axis. The anisotropy field (H_k) is given by:

$$H_k = \frac{2K_u}{M_s} \quad \text{[eq. S1.3]}$$

The total free energy (or energy landscape) can be written as:

$$E_{tot} = E_A + E_z = \frac{V_{eff}\, H_k M_s}{2} sin^2(\theta) - V_{eff} M_s H_a \sin(\theta + \alpha) \quad \text{[eq. S1.4]}$$

By evaluating the first and second derivatives of equation S1.4 with respect to θ, the free energy minima can be found for fixed values of H_a and α.

In order to generate a complete hysteresis loop, the applied field is first initialized to a value significantly larger than H_k, such that the energy landscape has only one (global) minimum. The equilibrium orientation (θ_{eq}) of the magnetization is unambiguously determined by the global minimum. As the applied field is changed, the energy landscape is re-evaluated for the new applied field, and the minima are calculated. The zero temperature approximation dictates that the new equilibrium orientation of the magnetization corresponds to the minimum closest to the previously occupied minimum. In other words, as the applied field is changed, the magnetization remains in the minimum previously occupied, provided that minimum still exists at the new applied field.

The components of the reduced magnetization in the direction of the applied field (m_x) and perpendicular to the applied field (m_y) are calculated as:

$$m_x = \frac{M_x}{M_s} = \cos\varphi = \sin(\theta_{eq} + \alpha) \quad \text{[eq. S1.5]}$$

$$m_y = \frac{M_y}{M_s} = \sin\varphi = \cos(\theta_{eq} + \alpha) \quad \text{[eq. S1.6]}$$

S2: Finite temperature Stoner-Wohlfarth model

To account for thermal fluctuations, the procedure outlined by Lanci and Kent [10] is followed. If a single minimum occurs in the energy landscape, the magnetization is assumed to lie in that minimum. If two minima exist, the number of the particles with magnetization in states 1 and 2 (n_1 and n_2, respectively) are governed by the two-state kinetic equation [17]:

$$\frac{dn_1}{dt} = -n_1 w_1 + n_2 w_2 \quad \text{[eq. S2.1]}$$

where

$$w_1 = f_o e^{\left(Eb_{1\to2}/k_B T\right)} \quad and \quad w_2 = f_o e^{\left(Eb_{2\to1}/k_B T\right)} \quad \text{[eq. S2.2a, S2.2b]}$$

and the barrier heights ($Eb_{1\to2}$ and $Eb_{2\to1}$) are computed from the energy landscape. The preexponential factor (f_o) which represents the Larmor frequency[18] is assumed to be independent of field and of order 10^9 Hz[18,19]. Integration of equation S2.1 (subject to the condition n_1+n_2=1) yields the expression for approach to thermal equilibrium:

$$n_1(t) = n_{1eq} + \left(n_1(0) - n_{1eq}\right)e^{\left(-t/\tau\right)} \quad \text{[eq. S2.3]}$$

where

$$n_{1eq} = \frac{w_2}{w_1 + w_2} \quad and \quad \frac{1}{\tau} = w_1 + w_2 \quad \text{[eq. S2.4a, S2.4b]}$$

Equation S2.3 is then used to calculate the fraction of magnetizations in state 1 (and therefore, the fraction in state 2), assuming a measurement time of 1 second[10]. The reduced, x and y-components of the magnetization are computed as the weighted sum of the magnetizations of the two states:

$$\bar{m}_x = n_1 m_{x1} + n_2 m_{x2} \qquad and \qquad \bar{m}_y = n_1 m_{y1} + n_2 m_{y2} \qquad \text{[eq.S2.5, S2.5b]}$$

While this derivation follows the procedure outlined by Lanci and Kent, we note that it is equivalent to the treatment of a finite temperature SW-model given by Ryabchenko and Kalita [20], which also assumes a two-level system and computes magnetic behavior based on a reduced temperature (T_{red}=$k_B T K_u V$, where K_u is the uniaxial anisotropy constant and V is the particle volume).

S3: Transverse remanence measurements

The transverse remanence is defined as the magnetization at zero applied field, perpendicular to the direction of the previously applied saturating field. The in-plane, transverse remanence of a Ni:$LiNbO_3$ sample has been measured as a function of the angle (α) of the applied field with respect to the hard axis. Measurements were performed using a commercial vector-VSM. The absolute value of reduced transverse remanence ($|M_{yR}/M_s|$) is shown in figure S3.1.

When the angle between the applied field and the hard axis (α) is greater than the angular distribution of the anisotropy axes, the films remain uniformly magnetized, even at zero applied field. The transverse remanence is greater than 98% of the saturation magnetization when the angle of the applied field with the hard axis is in the range $1^o \leq \alpha \leq 10^o$. When $|\alpha| < 1^o$, the transverse remanence drops to near zero, indicating that the sample is no longer uniformly magnetized and has broken up into domains [21]. The angular range over which the transverse remanence drop significantly is a measure of the angular distribution of the anisotropy axes [22]. For annealed nickel thin films on 128° Y-cut: $LiNbO_3$ the FWHM of the transverse remanence as a function of angle (α) is less than 0.5^o.

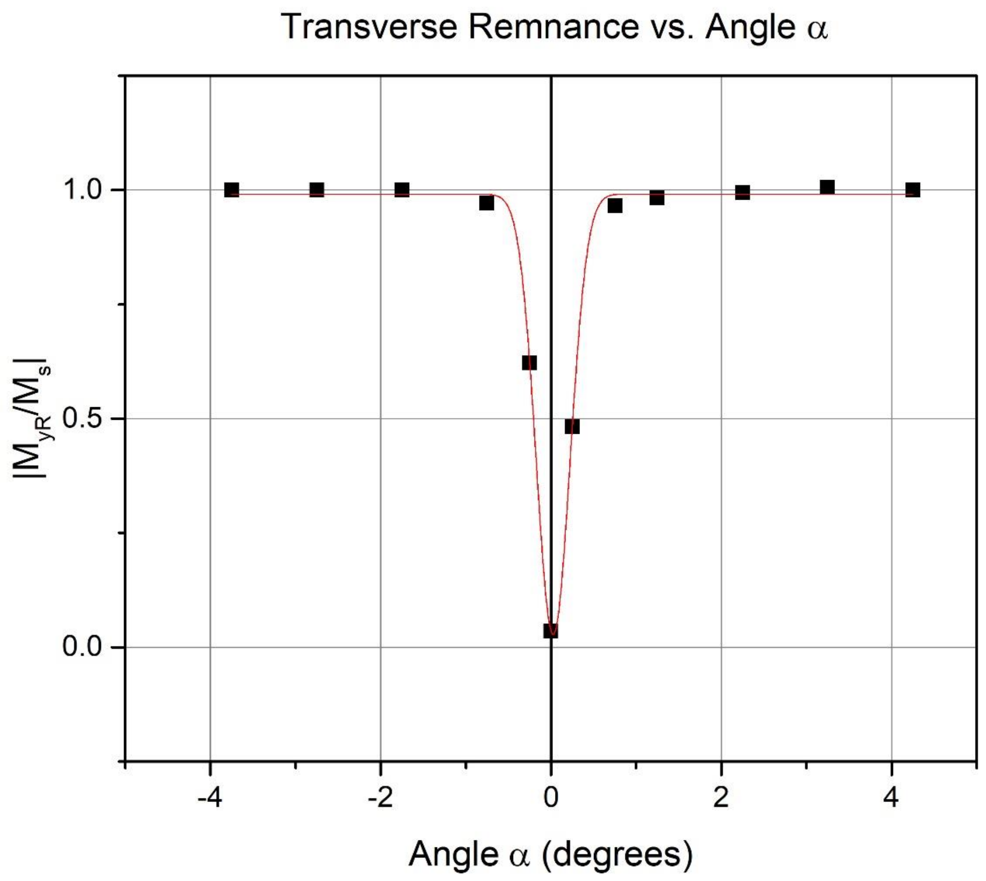

Figure S3.1: The absolute value of reduced transverse remanence as a function of the angle of the applied field with respect to the hard axis for a Ni:$LiNbO_3$ sample after low temperature anneal.

Figure S3.2(a-d) depicts a schematic representation of the process leading to the transverse remanence shown in figure S3.1. In figure S3.2 the anisotropy of the sample is assumed to be angularly distributed, with a distribution width of σ. The "fanning-out" of the easy (green) and hard (red) axes in figure S3.2 are a schematic representation of the distribution of anisotropy orientation from particle to particle. Figure S3.2(a) shows the saturating field applied close to the hard axis, but outside the distribution width σ. When the saturating field is removed, all magnetizations rotate to the closest easy direction, as shown in figure S3.2(b), and result in a remanence approximately equal to the saturation magnetization. In contrast, when the saturating field is applied on the hard axis, splitting the anisotropy distribution, as shown in figure S3.2(c), the rotations of the magnetizations to the nearest easy direction results in a remanence approximately equal to zero, as shown in figure S3.2(d).

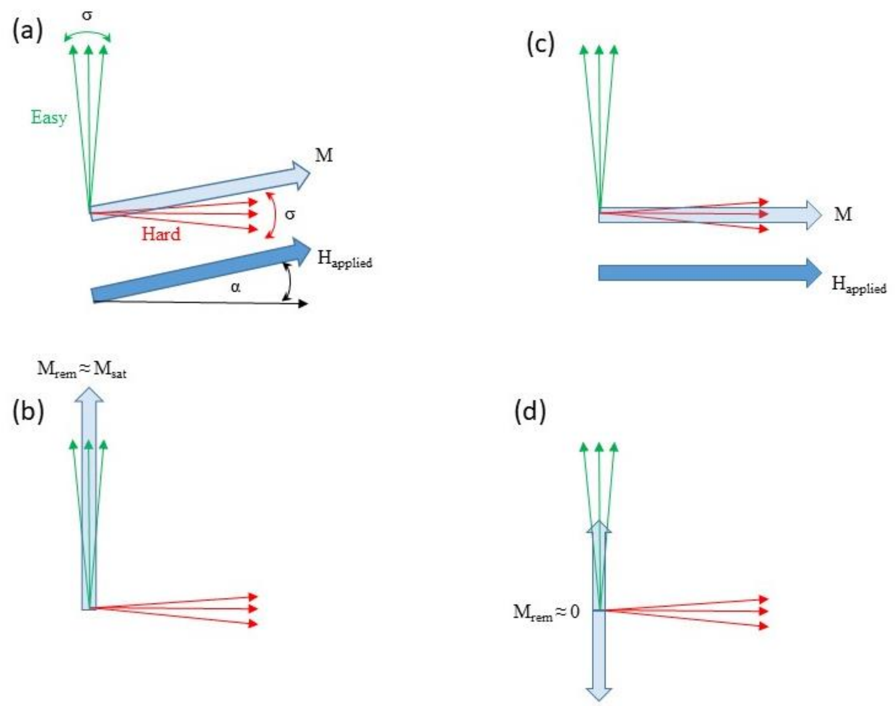

Figure S3.2(a-d): Schematic representation of magnetization to saturation and resulting transverse remanence for two angles of the applied field with respect to the hard axis.

S4: Numeric parameters for simulations

Table S4.1

**Variable**	**Description**	**Value**
T	Temperature in Kelvins	300 K
M_s	Saturation magnetization	55.1 emu/gm
H_k	Anisotropy field	520 Oe
σ_{Hk}	Anisotropy distribution width	20 Oe
V_{eff}	Effective particle volume	10^{-15} cm^3

All simulations in this work assume a temperature of 300 K and a saturation magnetization of 55.1 emu/gm [23]. The anisotropy field, anisotropy distribution width, and effective particle volume are adjusted in order to best fit the acquired data. The resulting values are, $H_k = 520$ Oe, $\sigma_{Hk} = 20$ Oe, and $V = 10^{-15}$ cm^3 and are in agreement with values estimated from other measurement techniques.
